# SNP-SNP interactions dominate the genetic architecture of candidate genes associated with left ventricular mass in african-americans of the GENOA study

**DOI:** 10.1186/1471-2350-11-160

**Published:** 2010-11-10

**Authors:** Kristin J Meyers, Jian Chu, Thomas H Mosley, Sharon LR Kardia

**Affiliations:** 1 Department of Population Health Sciences, University of Wisconsin, Madison, Wisconsin, USA; 2 Division of Biostatistics, Washington University, St. Louis, Missouri, USA; 3 Department of Medicine, University of Mississippi Medical Center, Jackson, Mississippi, USA; 4 Department of Epidemiology, University of Michigan, 4605 Washington Heights 4605 SPH-I, Ann Arbor, Michigan 48109-2029, USA

## Abstract

**Background:**

Left ventricular mass (LVM) is a strong, independent predictor of heart disease incidence and mortality. LVM is a complex, quantitative trait with genetic and environmental risk factors. This research characterizes the genetic architecture of LVM in an African-American population by examining the main and interactive effects of individual candidate gene single nucleotide polymorphisms (SNPs) and conventional risk factors for increased LVM.

**Methods:**

We used least-squares linear regression to investigate 1,878 SNPs from 234 candidate genes for SNP main effects, SNP-risk factor interactions, or SNP-SNP interactions associated with LVM in 1,328 African-Americans from the Genetic Epidemiology Network of Arteriopathy (GENOA) study. We reduced the probability of false positive results by implementing three analytic criteria: 1) the false discovery rate, 2) cross-validation, and 3) testing for internal replication of results.

**Results:**

We identified 409 SNP-SNP interactions passing all three criteria, while no SNP main effects or SNP-risk factor interactions passed all three. A multivariable model including four SNP-SNP interactions explained 11.3% of the variation in LVM in the full GENOA sample and 5.6% of LVM variation in independent test sets.

**Conclusions:**

The results of this research underscore that context dependent effects, specifically SNP-SNP interactions, may dominate genetic contributions to variation in complex traits such as LVM.

## Background

Heart disease, defined as myocardial infarction, hypertensive and ischemic heart disease, and heart failure, is the leading cause of mortality and morbidity in the United Sates [1]. Increased left ventricular mass (LVM) is a well-known, independent risk factor for heart disease incidence, mortality, and all-cause mortality [2-4]. LVM can be measured non-invasively via echocardiography and risk factors associated with increases in LVM include high blood pressure, high dietary salt intake, increased age, male gender, diabetes, and increased body mass index (BMI) [5-8]. African-Americans experience higher mean values of LVM and have almost twice the amount of left ventricular hypertrophy (clinical threshold for high LVM) compared to a non-Hispanic white population [5].

Family and twin studies have demonstrated that genetic factors significantly contribute to the inter-individual variation in LVM in numerous racial/ethnic groups. Heritability estimates range between 0.2 - 0.6 depending on the population being studied and risk factors adjusted for [9-12]. In an African-American population, the heritability of LVM, after adjustment for known risk factors, was estimated to be 0.46 [11]. As a follow-up to heritability studies, candidate gene association studies have attempted to test for associations with genetic variants in pathways involved in LVM. While some of the candidate gene results are promising, they have been limited by the lack of replication and the failure to consider the full spectrum of genetic effects involved in complex traits (ie. interactions). LVM is a complex, quantitative trait and by definition is the result of environmental factors, genetic factors, and interactions between. However, to date, most genetic association studies (candidate gene and genome wide) have inappropriately simplified genetic architecture by focusing on single SNP effects. Issues of failed replication are not surprising given that true genetic effects may not replicate in different study populations because they are specific to a given population, in a given environment or because the true architecture involves unaccounted interactions [13-16]. In order to fully understand the genetic architecture of complex traits such as LVM, single candidate gene SNP associations must be considered in the context of, and in conjunction with, environmental factors and other genetic variants.

The goal of this research was to explore the genetic architecture of LVM by identifying robust, replicated single SNP effects, SNP-environment interactions, and SNP-SNP interactions associated with LVM after adjusting for population stratification and relevant risk factors. In achieving this goal, we implemented a multi-stage approach that focuses on reducing the number of false-positive results and shows replication of effects within the study sample.

## Methods

### Study population

The National Heart Lung and Blood Institute established the Family Blood Pressure Program (FBPP) in 1996, joining established research networks investigating hypertension and cardiac diseases. One of the four networks in FBPP is the Genetic Epidemiology Network of Arteriopathy (GENOA), which recruited hypertensive African-Americans and non-Hispanic white sibships for linkage and association studies to investigate genetic contributions to hypertension and hypertensive target organ damage. Subjects for this particular GENOA sub-study were African-Americans recruited from Jackson, Mississippi. GENOA recruited sibships containing at least two individuals with clinically diagnosed essential hypertension before age 60. Hypertension was defined by a previous clinical diagnosis of hypertension by a physician with current anti-hypertensive treatment, or an average systolic blood pressure (SBP) ≥140 mmHg or diastolic blood pressure (DBP) ≥90 mmHg on the second and third clinic visit [17]. After identifying each hypertensive sibship, all members of the sibship were invited to participate regardless of their hypertension status. Exclusion criteria included secondary hypertension, alcoholism or drug abuse, pregnancy, Type I diabetes, or active malignancy. A total of 1,481 individuals were enrolled in GENOA. Informed consent for this study was obtained from all subjects and approval was granted by the institutional review board at the University of Mississippi Medical Center.

### Phenotype measurement

Data collection consisted of demographic information, medical history, clinical characteristics, lifestyle factors, and blood samples for genotyping and biomarker assays. Study visits were conducted in the morning after an overnight fast of at least eight hours. Blood pressure was measured with random zero sphygmomanometers and cuffs appropriate for arm size. Three readings were taken in the right arm after the participant rested in the sitting position for at least five minutes; the last two readings were averaged for the analysis. Height was measured by stadiometer, weight by electronic balance, and BMI was obtained by the standard calculation of weight (kg) divided by height squared (m^2^). Diabetes was considered present if the subject was being treated with insulin or oral agents or had a fasting glucose level ≥126 mg/dL. Smoking status was defined as self-described smoker within the past year. Use of anti-hypertensive medication was based on self-report during the clinical exam.

The outcome of interest, LVM, was derived using phased-array echocardiographs with M-mode, two-dimensional and pulsed, continuous wave, and colorflow Doppler capabilities. Standardized methods, along with training and certification, were used by field-center technicians to achieve high-quality recordings. Readings were performed at the New York Presbyterian Hospital-Weill Cornell Medical Center and verified by a single highly experienced investigator. The parasternal acoustic window was used to record at least 10 consecutive beats of two-dimensional and M-mode recordings of the left ventricular internal diameter (LVID) and wall thicknesses at, or just below, the tips of the anterior mitral leaflet in long- and short-axis views. Correct orientation of planes for imaging and Doppler recordings was verified using standardized protocols. Measurements were made using a computerized review station equipped with digitizing tablet and monitor screen overlay for calibration and performance of each measurement. LVID and interventricular septal and posterior wall thicknesses were measured using the two-dimensional view at end-diastole and end-systole according to the recommendations of the American Society of Echocardiography in up to three cardiac cycles [18]. Calculations of LVM were made using a necropsy-validated formula [19]. LVM has excellent reliability when measured through echocardiography; the correlation between repeated measures of LVM was 0.93 between paired echocardiograms in hypertensive adults [20]. LVM was measured on a total 1,440 African-American participants of GENOA.

### SNP selection and genotyping

One thousand nine hundred and fifty six SNPs from 268 genes known or hypothesized to be involved in blood pressure regulation, lipoprotein metabolism, inflammation, oxidative stress, vascular wall biology, obesity and diabetes were identified from the genetic association literature and positional candidate gene studies [21] to be genotyped in the entire GENOA population. SNPs were chosen based on a number of different criteria including the published literature, non-synonymous SNPs with a minor allele frequency (MAF) > 0.02, and tagSNPs identified using public databases such as dbSNP [22] and the SeattleSNPs database [23].

DNA was isolated using the PureGene DNA Isolation Kit from Gentra Systems (Minneapolis MN). Genotyping, based on polymerase chain reaction amplification techniques, was conducted at the University of Texas-Health Sciences Center at Houston using the TaqMan assay and ABI Prism^® ^Sequence Detection System (Applied Biosystems, Foster City CA). Primers and probes are available from the authors upon request. Quality control measures for genotyping assays included robotic liquid handling, separate pre- and post-PCR areas, standard protocols and quality control analyses including 5% duplicates, positive and negative controls, computerized sample tracking, and data validity checks. After these quality control procedures and removal of monomorphic SNPs, 1,878 SNPs from 234 genes were available for analysis in the African-American cohort of GENOA.(see Additional file 1) Primers and probes are available from authors upon request. Furthermore, FBPP data (including GENOA) is freely available to researchers upon request http://public.nhlbi.nih.gov/GeneticsGenomics/home/fbpp.aspx.

### Population substructure

The presence of population substructure is a concern for genetic epidemiological studies because the distribution of admixture proportions within a study sample can be a source of confounding, resulting in spurious SNP-disease associations [24-26]. Based on seventy-six microsatellite markers that were measured in both the GENOA cohort and also in the Human Genome Diversity Project (HGDP) [27], we used Structure to test for substructure in the GENOA African-American sample [28]. The populations that served as "parents" to the African-American cohort of GENOA in Structure analysis were the HGDP African Yoruba and Mandenka populations and the Caucasian GENOA population from Rochester, MN. After testing three possible underlying clusters in our data (K = 1, 2, or 3), Structure indicated that K = 2 clusters had the highest posterior probability. This indicates that given our data and the ancestral populations assumed there were no distinct underlying subgroups in our dataset, only admixture between African and European ancestors.

The underlying admixture within the African-American GENOA sample can be accounted for through principal component analysis (PCA) [29]. There were 453 microsatellite markers previously genotyped in GENOA for genome wide linkage analysis; these microsatellite markers were used to run PCA using R. Prior research has shown that association tests are not sensitive to the number of principle components included as long as a sufficient number of components are included in the model [30]. The first 20 principal components described approximately 20% of the underlying genetic variation and were used to adjust LVM using least-squares linear regression.

### Statistical analysis

Data analyses were conducted using the statistical language R (version 2.6) [31]. LVM was transformed using the natural logarithm in order to best approximate the distributional assumptions of linear regression. Allele and genotype frequencies were calculated using standard gene counting methods. Hardy-Weinberg equilibrium (HWE) was assessed using a chi-square test or Fisher's exact test if a genotype class had less than five individuals [32]. LVM was adjusted for risk factors including age, sex, SBP, height, weight, and admixture using least-squares linear regression. The residuals from the adjustment model were normally distributed, centered around zero, and used as the dependent variable for association tests. Tests for single SNP effects and SNP-SNP interactions utilized these residuals. For tests of SNP-covariate interactions, the respective variable was left out of the adjustment model and instead included in the model for interaction. For example when SNP-SBP interactions were tested, the LVM residuals were obtained by adjusting for age, sex, height, weight and admixture. Of the 1,440 African-Americans in GENOA with LVM measures, the final sample size for association analyses is 1,326 due to a limited number of individuals missing risk factor adjustment data, microsatellite data for PCA, or SNP data.

We used a multi-stage approach in order to identify both main and interactive genetic effects associated with adjusted logLVM. The first stage was dedicated to conducting association analyses for SNP effects, SNP-covariate interactions, and SNP-SNP interactions. The second stage focused on reducing the possibility of false-positive association results and replication of results within our GENOA sample. Finally, we conducted multivariable SNP modeling with associations passing the second stage of analysis. This analysis approach has been previously described by Kardia et al [33] and Smith et al [34].

### Stage I: Association analyses

In the first stage of analysis, we tested each of the 1,878 SNPs for association with adjusted logLVM using least-squares linear regression methods in the full sample [32,35]. The SNPs were modeled with two degrees of freedom, therefore assuming no underlying genetic model, and statistical significance for the main effect of each SNP was determined based on a likelihood ratio statistic.

Based on the 1,878 SNPs and 15 chosen covariates, all possible SNP-covariate interactions were assessed for association with adjusted logLVM using least-squares linear regression. The covariates considered in the interactions included age, sex, SBP, DBP, height, weight, diabetes status (0/1), hypertension status (0/1), use of anti-hypertensive medication (0/1), duration of hypertension, smoking status (0/1), myocardial infarction (0/1), total cholesterol, low density lipoprotein cholesterol (LDL), and triglycerides. Age, sex, SBP, height, and weight were left out of the adjustment model in order to include this main effect in the respective test for interaction. We determined significance of the SNP-covariate interaction with a likelihood ratio test statistic comparing a full model (including interaction terms and main effects of the variables in the interaction term) to a reduced model that contains the main effects of the covariate and SNP being tested.

All possible pairwise SNP-SNP interactions were tested with SNPs coded as two dummy variables to allow testing for all possible statistical epistatic effects [36]. The statistical significance of the SNP-SNP interaction was based on a likelihood ratio test comparing the full model including all interaction terms to a reduced model with only the main effects of each SNP (up to four degrees of freedom depending on presence of all genotypic combinations) [36].

### Stage II: Reduction of false positive associations

The second stage of analysis was focused on reducing the possibility of false-positive association results and replication of results within our GENOA sample. We did this by implementing three analytic approaches: 1) False Discovery Rate (FDR) [37], 2) four-fold cross-validiation (CV) [38], and 3) internal replication of results between two subsets of the data. Only associations passing the pre-determined thresholds for all three approaches were considered positive associations.

The first step for reducing the probability of false positive results was to calculate the FDR q-value for all association tests [37]. FDR is a method that controls for the proportion of "rejected hypotheses" that are rejected falsely. For the single SNP associations, the vector of model p-values was used to calculate the q-value, while for the SNP-covariate and SNP-SNP interactions, the vectors of partial F test p-values were used to calculate the q-value. An FDR q-value threshold <0.30 was used to determine significance.

The second approach for minimizing false positive results was to use four-fold CV, a method that reduces false positive results by eliminating associations that lack predictive ability in independent test samples. We performed CV by dividing the full sample into four equally sized groups. Three of the four groups were combined into a training dataset, and the modeling strategy outlined above was carried out to estimate model coefficients. These coefficients were then applied to the fourth group, the testing dataset, to make predictions about the value of the outcome variable of each individual in the independent test sample. This process was repeated for each of the four testing sets. Because random variations in the sampling of the four mutually exclusive test groups can potentially impact the estimates of CV R^2^, this procedure was repeated ten times and the CV R^2 ^values were averaged [38]. Single SNP associations were considered cross-validated if the average percent variation predicted in independent test samples (CV R^2^) was greater than 0.5% and interactions were considered cross-validated if the difference in average percent variation predicted in independent test samples between the full model containing the interaction terms and the reduced model containing only main effect terms was greater than 0.5%. This threshold of 0.5% was chosen because permutation tests on the models investigated in this paper, we found that the probability of observing a CV R^2 ^× 100 greater than 0.5% by chance alone was less than 5% (results not shown). That is, Pr(CV R^2 ^× 100 > 0.5%) <0.05 under the null hypothesis of no association.

The third and final step to reduce false positive results was to demonstrate replication of effects within our GENOA sample. Considering the entire sample of African-Americans and randomly sampling one sibling from each sibship, without replacement, the first replication subset sample was created. From the remaining people, we randomly sampled a second sibling from each sibship to establish the second sample. Association analyses were then conducted in both of the subset samples. If a SNP, SNP-covariate, or SNP-SNP association replicated across these two samples (α = 0.10), passed FDR and CV criteria in the full sample, it was tested for homogeneity of direction and magnitude of effect across the two samples.

### Multivariable SNP modeling

Based on the association tests that passed all three of the above criteria (FDR q-value < 0.30, replication in replication datasets with α = 0.10, and CV R^2 ^> 0.005), we built a multivariable linear regression model using forward selection in the full sample of GENOA African-Americans. Residuals from the age, sex, SBP, height, weight, and admixture adjusted logLVM were used as the dependent variable for this multivariable model. The increase in percent variation of adjusted LVM explained was then calculated, as was the increased predictive ability of the model based on the full model CV R^2 ^with the addition of each term. Because the full sample of individuals contains siblings, the associations that were included in the final multivariable model were also tested using a linear mixed effects model to account for the familial correlation and ensure that the results were not dependent upon the underlying familial correlation in the data.

## Results

To examine the genetic architecture of LVM in African-American individuals, we used data from the GENOA study for association analysis. In general, this is an older (mean age 63) hypertensive cohort (79% hypertensive) with an average BMI of 31 and 29% diabetic (Table 1). The average LVM is 160.8 grams.

**Table 1:** Descriptive statistics for the full African-American cohort of GENOA and two internal replication subset samples.

		Full Sample		Subset 1		Subset 2
Variable	N	Mean ± St. Dev.	N	Mean ± St. Dev.	N	Mean ± St. Dev.
Age, years.	1328	62.7 ± 9.5	491	62.99 ± 9.63	496	63.09 ± 9.62
BMI, kg/m^2^	1326	31.5 ± 6.6	488	31.67 ± 7.01	494	31.5 ± 6.88
SBP, mmHg	1328	138.3 ± 21.1	491	139.3 ± 21.49	496	138.5 ± 20.77
DBP, mmHg	1328	79.6 ± 10.8	491	80.28 ± 10.76	496	79.92 ± 11.35
Height, cm	1326	168.4 ± 8.8	488	169.4 ± 9.15	494	169.2 ± 9.08
Weight, kg	1326	89.3 ± 19	488	90.66 ± 19.83	494	90.01 ± 19.5
Duration of hypertension, years	1046	16.5 ± 12.8	404	16.79 ± 13.24	396	16.17 ± 12.49
LV Mass, g	1328	160.8 ± 47.1	477	167.4 ± 51.66	477	163.5 ± 46.42
Sex, male	1328	393 (29.6%)	491	187 (38.1%)	496	175 (35.3%)
Smoker	1328	188 (14.2%)	491	78 (15.9%)	496	79 (15.9%)
Diabetic	1328	387 (29.1%)	491	150 (30.5%)	496	144 (29.0%)
LV Hypertrophy	1328	210 (15.8%)	491	90 (18.3%)	.496	81 (16.3%)
Hypertensive	1328	1,046 (78.8%)	491	400 (81.5%)	496	391 (78.8%)
Use anti-hypertensive medication	1328	930 (70.0%)	491	357 (72.7%)	496	344 (69.4%)

BMI = body mass index, SBP = systolic blood pressure, DBP = diastolic blood pressure, LV = left ventricular

Smoker: Self-reported smoker within the past year.

Diabetic: current treatment with insulin or oral agents OR a fasting glucose ≥126 mg/dL.

LV Hypertrophy: sex-specific thresholds; LVMI ≥51 g/m^2.7 ^for males, LVMI ≥49 g/m^2.7 ^for females

Hypertensive: previous clinical diagnosis by physician with current anti-hypertensive treatment, OR an average SBP ≥140 mmHG or DBP ≥90 mmHG on the second and third clinic visits.

### Stage I and II results

1,878 SNPs were tested for association with adjusted logLVM. Of these, 221 had a p-value < 0.10 in the full sample, the minimum p-value was 9.24 × 10^-4 ^(SNP: rs12460421, FDR q-value = 0.738, CV R^2 ^= 0.0033). None of these single SNP associations had an FDR q-value < 0.30 and only one had a CV R^2 ^> 0.005 (SNP: rs2182833). Table 2 summarizes the number of results passing each of the three pre-determined multiple testing criteria for the SNP main effects, the SNP-covariate interactions, and the SNP-SNP interactions.

**Table 2:** Summary of the number of associations passing each of the three multiple testing criteria.

	SNP Main Effects	SNP-Covariate Interactions	SNP-SNP Interactions
Total # of Tests	1878	28075	1740614
P-value < 0.10*	221	3217	192202
FDR q-value <0.30	0	10	3083
Cross-Validation R^2 ^>0.005	1	112	5007
Replication (P < 0.10 both groups)	14	303	17593
FDR + CV + Replication	0	0	409

This table outlines the number of associations (single SNP, SNP-covariate interactions, and SNP-SNP interactions) passing each level of multiple testing criteria (False Discovery Rate (FDR), 4-fold cross validation (CV) repeated and averaged 10 times, and internal replication in two subsets of the full dataset). The intersection of associations passing all three criteria reveals little overlap.

*P-values for SNP main effects are from a 2 degree of freedom likelihood ratio test statistic. The SNP-covariate and SNP-SNP interactions p-values were determined from a likelihood ratio test comparing a full model (including all interactions and main effects) to a reduced model only containing main effects of covariates and/or SNPs.

 There were a total of 28,075 SNP-covariate interactions tested. Ten of those had an FDR q-value < 0.30 (p-values ranging from 1.95 × 10^-6 ^to 9.59 × 10^-5^), 303 replicated across sample subsets, and 112 had a CV R^2 ^> 0.005. However, none of the SNP-covariate interactions passed all three criteria.

Based on the 1,878 SNPs, all possible SNP-SNP interactions were tested for a total of 1,740,614 associations. 409 of these associations passed all three criteria with an FDR q-value < 0.30, replicating in both subsets of the data, and had a CV R^2 ^> 0.005. The interaction with the lowest partial F-test p-value in the full sample was rs17876148*rs12971616 (p-value = 4.35 × 10^-8^, FDR q-value = 0.0139, CV R^2 ^= 0.0219).

### Multivariable modeling results

A multivariable model was built to determine if a significant proportion of the variation in LVM could be explained by the joint effect of these SNPs and their interactions. To avoid over-parameterizing the model, only four SNP-SNP interactions were chosen for the final multivariable model. The model building process began with the interaction with the most significant likelihood ratio test statistic p-value in the full sample (rs35314437*rs7552841) (first row of Table 3). A forward selection process was implemented with the remaining top nine SNP-SNP interaction models. At each decision point, the SNP-SNP interaction resulting in the lowest likelihood ratio test statistic p-value for including main and interaction SNP effects was added to the model. Table 3 shows the detailed association results for the ten most significant SNP-SNP interaction models considered in the forward selection process. Ultimately the following four interactions, and their main effects, were included in the final multivariable model in the order listed: rs35314437*rs7552841, rs257376*rs5267, rs17876148*rs12971616, and rs6745660*rs12460421 (bold rows in Table 3). Combined, these interactions explained 11.3% of the variation in logLVM after adjustment for age, sex, SBP, height, weight, and admixture. Table 4 outlines the variation in LVM explained by the addition of the main and interactive effects of each SNP-SNP interaction. The predictive ability of the model increases steadily with the addition of each interaction term as indicated by the increase in CV R^2^. CV R^2 ^was 5.56% when the full model included all four SNP-SNP interactions. Detailed mathematical description of the final model which includes main and interactive effects is included as an additional file.(see Additional file 2) Finally, these inferences are robust to family structure. When each of the four SNP-SNP interactions were tested using linear mixed effects models to account for familial correlation, the p-values from the least squares linear regression and linear mixed effects models had a Pearson correlation coefficient >0.99.

**Table 3:** Detailed results for the ten most significant SNP-SNP interaction models.

SNP 1	SNP 2	DF* for Interaction Test	Interaction P-value in full sample	Model P-value in full sample	Interaction q-value in full sample	CV* R^2 ^in full sample	Interaction P-value (Sample 1)	Interaction P-value (Sample 2)
rs35314437	rs7552841	2	1.78 × 10^-7^	3.88 × 10^-8^	0.0142	0.0165	0.0202	4.21 × 10^-6^
rs257376	rs5267	3	1.33 × 10^-6^	9.11 × 10^-8^	0.0218	0.0031	0.0965	0.0442
rs2229169	rs6664855	4	2.45 × 10^-7^	1.19 × 10^-7^	0.0142	0.0094	0.0004	0.0028
rs10482839	rs7552841	3	2.13 × 10^-6^	2.78 × 10^-7^	0.0256	0.0143	0.0276	3.96 × 10^-5^
rs17876148	rs12971616	4	4.35 × 10^-8^	3.14 × 10^-7^	0.0139	0.0151	1.85 × 10^-7^	0.0389
rs936211	rs521898	2	1.17 × 10^-6^	1.07 × 10^-6^	0.0211	0.0115	0.0663	0.0023
rs6745660	rs12460421	4	0.0002	1.09 × 10^-6^	0.2247	0.0158	0.0856	0.0054
rs945032	rs12028945	4	6.45 × 10^-6^	1.11 × 10^-6^	0.0385	0.0103	0.0028	0.0011
rs17876144	rs12971616	4	7.29 × 10^-8^	1.14 × 10^-6^	0.0139	0.012	2.73 × 10^-6^	0.0341
rs35314437	rs4846052	1	1.73 × 10^-7^	1.15 × 10^-6^	0.0142	0.0177	0.0021	4.14 × 10^-5^

Table 3 outlines the detailed association and multiple testing results for the top ten SNP-SNP interactions passing all three multiple testing criteria. "Interaction p-values" are from an up to 4 degree of freedom likelihood ratio test (depending on number of genotype classes represented in GENOA sample), "model p-value" column is from the likelihood ratio for the model including main effects and interactions compared to a null model (up to 8 degrees of freedom), q-value was assessed from the "interaction p-value", CV R^2 ^is the difference in CV R^2 ^when the interaction terms are included in the CV process compared to only main effects of SNPs, and the final two columns are the interaction p-values for the internal replication subset samples. These ten models were used in the multivariable model building process with the bold rows indicating interactions included in final model.

*DF = degrees of freedom, CV = cross-validation

**Table 4:** Outline of model improvement with addition of each SNP-SNP interaction included in final multivariable model.

Model	Interaction Terms in Model	Total # of Terms in Model	R^2^	Adjusted R^2^	LR* p-value for Additional Terms	Full Model CV* R^2^
1	(rs35314437 * rs7552841)	5	0.034	0.03	n/a	0.0165
2	Model 1 + (rs257376 * rs5267)	12	0.073	0.064	2.094 × 10^-8 ^(df = 7)	0.0332
3	Model 2 + (rs17876148 * rs12971616)	20	0.108	0.093	2.208 × 10^-7 ^(df = 8)	0.046
4	Model 3 + (rs6745660 * rs12460421)	28	0.133	0.113	3.631 × 10^-5 ^(df = 8)	0.0556

A multivariable model including a total of four SNP-SNP interactions was built in the African-American cohort of GENOA using forward selection. With the addition of each SNP-SNP interaction, along with each SNP's respective main effect, the variability in adjusted logLVM increased (assessed by adjusted R^2^) as did the predictive ability of the model in cross-validation test sets (assessed by full model CV R^2^). The final multivariable model explained 11.3% of the observed inter-individual variation in adjusted logLVM in GENOA and increased the predictive ability of the model by 5.6%.

* LR = likelihood ratio, CV = cross-validation

## Discussion

LVM is a complex, quantitative trait highly predictive of incident heart disease. While many studies have investigated candidate gene associations with LVM, to our knowledge, no one has investigated the spectrum of candidate gene effects for association with LVM including SNP main effects, SNP-covariate interactions, and SNP-SNP interactions. Our motivating hypothesis was that variations within positional and functional candidate genes for hypertension and heart disease are associated with LVM via interactive effects, in addition to single SNP effects. In examining this hypothesis, we demonstrated SNP-SNP interactions dominate the genetic architecture of LVM in the African-American cohort of GENOA.

One notable aspect of these results is the overwhelming presence of statistically significant epistasis in the absence of marginal SNP effects. There has been debate in the literature about the best way to test for interactions while minimizing computational burden and the possibility of false positives [39,40]. One strategy is to condition tests for SNP-SNP interactions on at least weakly significant marginal SNP effects (ex. p-value < 0.10) [39]. While this method will reduce the number of tests conducted, not all SNP-SNP interactions are expected to demonstrate marginal effects [40]. Many previous studies have identified epistasis in the absence of main effects. One example was found in dyslipidemia; individually, none of the three SNPs within the USF1 gene tested for association with various lipid measures showed any significance [41]. However, significant interactions between SNPs in USF1 and SNPs in HSL and APOC3 were identified as significantly associated with triglycerides and apoE levels [41]. Additional examples of epistasis in the absence of main effects in heart disease traits are found in atrial fibrillation [42] and coronary artery disease [43]. An additional case against conditioning searches for interaction based on initially significant main effects is the possible bias from the "winners curse", a type of ascertainment bias that describes the first positive report of a genetic variant overestimating the true effect size. Follow-up searches for interaction based on this overestimated effect tend to be underpowered [44,45]. Likewise, our results do not support conditioning searches for interaction on main effects. Of the eight SNPs included in the multivariable model, the range of main effect SNP p-values was 9.24 × 10^-4 ^(rs12460421) to 0.415 (rs12971616) (Table 5). Conditioning searches for interaction on main effects would have precluded investigation of two of the four robust interactions included in the final multivariable model. This conclusion is directly parallel with a recent study demonstrating the feasibility and justification of genome wide interaction searches without conditioning on main effects [46].

**Table 5:** Positional and functional details of SNPs included in the final multivariable model.

SNP	Gene	Chromo-some	Position	Minor Allele	Minor Allele Freq.	Type	Biological Processes*	HWE p-value	P-value for Main Effect of SNP
rs35314437	MPO	17q	53704206	G	0.015	Synonymous	Response to oxidative stress, anti-apoptosis	1	0.044
rs7552841	PCSK9	1p	55291340	A	0.237	Intron	Cholesterol homeostasis & metabolic processes	0.6532	0.0215
rs257376	PRKAR2B	7q	106393948	A	0.486	Synonymous	Intra-cellular signaling cascade	0.5951	0.0512
rs5267	NPPC	2q	232615776	T	0.196	Non-synonymous	Regulation of BP & vasoconstriction	0.7323	0.0067
rs17876148	PON2	7q	94877484	A	0.093	Intron	None reported	0.0017	0.1564
rs12971616	CARM1	19p	10875937	A	0.13	Intron	Transcription regulation, histone methylation	0.1464	0.4149
rs6745660	HSPD1	2q	198057781	G	0.351	3' near gene	Protein folding, response to stress	0.0625	0.0188
rs12460421	CARM1	19p	10842352	G	0.438	5' near gene	Transcription regulation, histone methylation	0.6796	9.24 × 10^-4^

* Biological process of the gene are a subset of those reported in the Michigan Molecular Interactions website: http://mimi.ncibi.org/MimiWeb/main-page.jsp

A concern for the occurrence of type I errors in the face of so many hypothesis tests is substantial and valid. Genetic association studies in the literature have suffered from a great lack of replicability. This lack of replication can be attributed to various reasons. Some might be due to population specific effects resulting from differing allelic and environmental distributions in various geographical regions, false positive reports, or overestimated initial effects (the "winners curse"). Recognizing that replication in an independent cohort might not be possible because of various sources of heterogeneity; we sought to find genetic associations that replicated within our study sample and were robust across numerous multiple testing adjustment methods. The relative low level of agreement between results filtered through FDR, internal replication, and CV supports the conservative nature of our strategy for determining which results are robust and significant. Furthermore, a similar analysis approach applied to two different phenotypes, ankle brachial index [33] and leukoaraiosis [34], identified different patterns of genetic architecture, with less emphasis on SNP-SNP interactions. Therefore, we feel this analysis approach is useful for the reduction of type I errors and may provide a tool for identifying unique patterns of genetic architecture, which are likely to vary based on the phenotype of study.

A natural question arising from our study results is how these SNPs interact biologically? As these SNPs were selected from "candidate genes", biological plausibility can be argued for any individual SNP. Table 5 outlines positional and functional information for each SNP. Inferences of protein-protein interactions are more difficult to make from this research because statistical tests for SNP-SNP interactions will not necessarily mirror tests for biological interactions [36]. We searched the Michigan Molecular Interactions database [47] and PubMed [48] for any previously reported protein interactions between the four pairwise gene interactions in the multivariable model. No protein interactions were identified in those databases for the gene combinations reported in Table 5. This is not surprising as making the connection between statistical epistasis and biological epistasis is difficult and arguably not permissible [36,49]. Furthermore, since association testing relies on the concept of linkage disequilibrium, it is always possible at least one of the "causal" SNPs is in a different gene than the reported gene, and therefore we would not expect to see the biological interaction between reported genes. Despite these caveats, the strength and concordance of the associations detected in both traditional hypothesis testing methods (ie. FDR and internal replication) and prediction testing methods (ie. CV) gives us confidences in the effects these SNP-SNP interactions have on LVM. Of particular potential biological relevance is the MPO SNP (rs35314437) that was identified in the first interaction term included in our multivariable model. Work done by Vasilyev et al found that MPO-generated oxidants have a profound, adverse effect on left ventricular remodeling and function [50]. Further, Ng et al concluded that MPO biomarkers increased the specificity of n-terminal pro-B-type natriuretic peptide as a screening tool for identifying undiagnosed left ventricular systolic dysfunction [51]. An interesting future direction for research would be to further pursue how the effects of MPO on left ventricular structure and function may be modified by other genes such as PCSK9.

## Conclusions

There is much yet to be understood about LVM and why it is so highly predictive of heart disease and all-cause mortality, independent of other cardiovascular risk factors [4]. The results of this research underscore the biological complexity underlying LVM and that context dependent effects, specifically SNP-SNP interactions, may dominate the genetic architecture of LVM. In this study we focused on main and interactive genetic effects of SNPs within candidate genes. Given the complexity of LVM and replication issues inherent for heterogeneous traits, we demonstrate a conservative approach for identifying robust associations within a given population. Future examinations into the genetic architecture of LVM should include replication efforts of the interactions reported in independent populations, with detailed consideration of sources of heterogeneity such as differing allele frequencies and population characteristics.

## Competing interests

The authors declare that they have no competing interests.

## Authors' contributions

SLRK and KJM conceived of and designed the study. KJM and JC conducted statistical analysis. THM was responsible for recruiting and data collection at the Jackson field center of GENOA. KJM prepared and wrote the manuscript. All authors were involved in critical revisions of the manuscript and have agreed upon its final content.

## Pre-publication history

The pre-publication history for this paper can be accessed here:

http://www.biomedcentral.com/1471-2350/11/160/prepub

## Supplementary Material

Additional file 1 **Contains a list of all SNPs (and their respective gene) investigated**. Click here for file

Additional file 2 **Contains the R output for the multivariable model including 4 SNP-SNP interactions**. Click here for file
