# Supplementary material for: SNP-SNP interactions dominate the genetic architecture of candidate genes associated with left ventricular mass in african-americans of the GENOA study
Source: BMC Med Genet. 2010 Nov 10;11:160. doi: 10.1186/1471-2350-11-160 (PMC2991303; doi:10.1186/1471-2350-11-160)
Supplement: Additional file 2 — Contains the R output for the multivariable model including 4 SNP-SNP interactions. [file 1471-2350-11-160-S2.PDF]

Final Multivariable Model Including Main and Interactive Effects of SNPs

Call:
```
lm(formula = outcome ~ (rs35314437 * rs7552841) + (rs257376 * rs5267)
    + (rs17876148 * rs12971616) + (rs6745660 * rs12460421),
   data = temp.data, na.action = na.omit)
```

Residuals:

Min	1Q	Median	3Q	Max
-0.830603	-0.135538	-0.007787	0.127133	0.780865

Coefficients: (1 not defined because of singularities)

	**Estimate**	**Std. Error**	**t value**	**Pr(>|t|)**
(Intercept)	-0.1180372	0.2120282	-0.557	0.577833
rs35314437AG	0.8119519	0.1475859	5.502	4.60e-08 ***
rs7552841AG	-0.0565045	0.0290478	-1.945	0.051983 .
rs7552841GG	-0.0575226	0.0283443	-2.029	0.042636 *
rs257376AG	-0.0000235	0.0154260	-0.002	0.998785
rs257376GG	-0.0455340	0.0173737	-2.621	0.008883 **
rs5267CT	-0.0462570	0.0456031	-1.014	0.310627
rs5267TT	0.0262561	0.1452409	0.181	0.856574
rs17876148AG	0.0669107	0.2271222	0.295	0.768349
rs17876148GG	0.1590737	0.2126924	0.748	0.454665
rs12971616AG	0.8133661	0.2325470	3.498	0.000487 ***
rs12971616GG	0.1181882	0.2172383	0.544	0.586510
rs6745660AG	0.0427028	0.0222589	1.918	0.055292 .
rs6745660GG	-0.0249412	0.0319189	-0.781	0.434727
rs12460421AG	0.0381558	0.0211493	1.804	0.071465 .
rs12460421GG	0.0546251	0.0260797	2.095	0.036422 *
rs35314437AG:rs7552841AG	-0.6559831	0.1576387	-4.161	3.39e-05 ***
rs35314437AG:rs7552841GG	-0.8125350	0.1541850	-5.270	1.62e-07 ***
rs257376AG:rs5267CT	0.0205727	0.0544763	0.378	0.705762
rs257376GG:rs5267CT	0.1974938	0.0615307	3.210	0.001364 **
rs257376AG:rs5267TT	1.0189625	0.2499302	4.077	4.86e-05 ***
rs257376GG:rs5267TT	NA	NA	NA	NA
rs17876148AG:rs12971616AG	-0.7015621	0.2513903	-2.791	0.005343 **
rs17876148GG:rs12971616AG	-0.8486968	0.2361785	-3.593	0.000340 ***
rs17876148AG:rs12971616GG	-0.0445636	0.2351060	-0.190	0.849697
rs17876148GG:rs12971616GG	-0.1276900	0.2209328	-0.578	0.563401
rs6745660AG:rs12460421AG	-0.0330628	0.0288780	-1.145	0.252474
rs6745660GG:rs12460421AG	-0.0305110	0.0406347	-0.751	0.452884
rs6745660AG:rs12460421GG	-0.0563987	0.0358342	-1.574	0.115781
rs6745660GG:rs12460421GG	0.2109716	0.0608447	3.467	0.000544 ***

Signif. codes: 0 ‘***’ 0.001 ‘**’ 0.01 ‘*’ 0.05 ‘.’ 0.1 ‘ ’ 1

Residual standard error: 0.2034 on 1192 degrees of freedom
Multiple R-squared: 0.1331, **Adjusted R-squared: 0.1127**
F-statistic: 6.534 on 28 and 1192 DF, **p-value: < 2.2e-16**
